# Structural Basis of VSIG3: The Ligand for VISTA

**DOI:** 10.3389/fimmu.2021.625808

**Published:** 2021-03-25

**Authors:** Xiaoxue Xie, Caiping Chen, Wenting Chen, Jingwei Jiang, Lanlan Wang, Tingting Li, Hongbin Sun, Jun Liu

**Affiliations:** ^1^ New Drug Screening Center, China Pharmaceutical University, Nanjing, China; ^2^ Jiangsu Key Laboratory of Drug Discovery for Metabolic Disease, China Pharmaceutical University, Nanjing, China

**Keywords:** VISTA, VSIG3, X-ray, crystal structure, drug discovery

## Abstract

B7 family members and their receptors play key roles in regulating T cell responses, and constitute very attractive targets for developing immunotherapeutic drugs. V-Set and Immunoglobulin domain containing 3 (VSIG3), a ligand for the novel B7 family immune checkpoint V-domain immunoglobulin suppressor of T cell activation (VISTA), can significantly inhibit T cell functions. Inhibitors targeting the VISTA/VSIG3 pathway are of great significance in tumor immunology. Here, we show the crystal structure of the extracellular domain (ECD) of the human VSIG3 protein at 2.64 angstrom resolution, and we produce recombinant human VSIG-3 ECD in both CHO cells and *E. coli*. Furthermore, we demonstrated the interaction of VISTA and VSIG3 by coimmunoprecipitation (Co-IP). Based on protein-protein docking for VISTA and VSIG3, we report a small molecule inhibitor of VSIG3 K284-3046 and evaluate its biological activities *in vitro*. This study was the first to reveal the crystal structure of VSIG3, and provides the structural basis for designing antibodies or compounds for the unique VSIG3/VISTA coinhibitory pathway in the treatment of cancers, autoimmune diseases and may be beneficial of designing vaccines.

## Introduction

VSIG3 (V-set and immunoglobulin domain containing 3) is a member of the immunoglobulin superfamily (IgSF), which is also called the immunoglobulin superfamily 11 gene (IgSF11), and it is highly expressed in the brain and testis (1). VSIG3 is a type I transmembrane protein, and the extracellular domain contains a V-type and a C2-type immunoglobulin domain and a C-terminal PDZ domain. The protein exhibits high homology with coxsackie-adenovirus receptor (CAR), endothelial cell-selective adhesion molecule (ESAM) and CXADR-like membrane protein (CLMP) and functions as a cell adhesion molecule (2, 3). With respect to other functions, VSIG3 regulates the proliferation and differentiation of cerebellar granule cell precursors (CGCPs) (4). The absence of VSIG3 results in the loss of the integrity of the blood-testis barrier (5). In addition, VSIG3 acts as a dual binding partner for postsynaptic scaffold protein PSD-95 and AMPA glutamate receptor to regulate excitatory synaptic transmission and plasticity (6, 7). In zebrafish, VSIG3 mutations affect the migration and survival of melanin and its precursor proteins (8, 9). VSIG3 is minimally expressed in normal tissues but is significantly upregulated in intestinal-type gastric cancer, colorectal cancer and hepatocellular carcinoma, indicating that the protein is important as a tumor-associated antigen (TAA) for clinical applications in tumor immunotherapy (10). The polypeptide vaccine designed and synthesized based on VSIG3 activates specific cytotoxic T lymphocytes (CTLs) to kill tumor cells and improve the survival rate of gastric cancer patients (10).

V-domain immunoglobulin suppressor of T cell activation (VISTA) is an immune checkpoint protein that belongs to the immunoglobulin family and interacts with unknown proteins as ligands or receptors to generate cosuppressor signals that regulate T cell functions (11). VISTA is highly expressed in myeloid cells (i.e., CD11b^+^, macrophages and MDSCs) and T cells that infiltrate tumors (12–15). In addition, VISTA is expressed on naive T cells and essential for maintaining peripheral tolerance (16). The absence of VISTA aggravates the development of autoimmune diseases, such as experimental autoimmune encephalomyelitis (EAE), mouse systemic lupus erythematosus and allergic asthma (17–19). VISTA is highly expressed in a variety of cancers, such as gastric cancer, oral squamous cell carcinoma, non-small-cell lung cancer, ovarian cancer and colorectal cancer (20–24). In a study of pancreatic cancer, researchers found that VISTA was preferentially expressed at higher levels than PD-L1 (13). In addition, the effects of VISTA and PD-L1 on T cells are nonredundant (25). Thus, VISTA plays an important role in regulating tumors and autoimmune diseases. Understanding the binding mode of VISTA at the molecular level is essential for discovering drugs targeting the VISTA pathway.

In 2017, VSIG3 was reported to be the specific binding partner of VISTA. Researchers constructed a mammalian cell-based expression library that displayed the extracellular segment of all human single-transmembrane proteins, and VSIG3 was screened to bind to VISTA. The binding of VISTA and VSIG3 was dose-dependent and could be blocked by a VISTA antibody or VSIG3 antibody (26). In 2018, Wang et al. showed that VSIG3 could represent a VISTA ligand that significantly inhibited the function of T cells through a novel VSIG3/VISTA pathway (27). Johnston et al. reported that the extracellular domain of VISTA is uniquely rich in histidine residues, which makes the domain positively charged (28). These researchers hypothesized that histidine-rich regions create pH-dependent binding sites, which preferentially bind to receptors in acidic tumor microenvironments. Then, they performed a ligand-based receptor capture assay with VISTA-Fc chimeric protein at pH 6.0 and identified P-selectin-glycoprotein ligand-1 (PSGL-1) as a VISTA receptor (29). PSGL-1 binding to VISTA is mediated by charged interactions between sulfated tyrosine and protonated histidine residues. However, investigators did not demonstrate any *in vivo* interactions between VISTA and PSGL-1; therefore, the physiological relevance of this complex remains speculative (30).

Here, we provide the crystal structure of the human VSIG3 ECD and characterized the interaction of VISTA with its ligand VSIG3 by Co-IP. Based on protein-protein docking for VISTA and VSIG3, we report a small molecule inhibitor of VSIG3 termed K284-3046 and evaluate its biological activities *in vitro*.

## Method and Materials

### The Expression and Purification of Human VSIG3 Protein From *E. coli*

The gene encoding human VSIG3 (residues 23–241; UniProt: Q5DX21) was cloned into pET28a. Proteins were expressed in Escherichia coli BL21 (DE3). Cells were cultured in LB at 37°C. The protein production was induced with 0.5 mM IPTG at OD600 of 1.0 and the cells were cultured for additional 5h. The cells were collected, sonicated and centrifuged to obtain inclusion body proteins. Proteins were washed twice with 25 mM Tris-HCl pH 8.0 containing 150 mM NaCl, 0.5% Triton X-100 and once more with the same buffer without detergent. The inclusion bodies were solubilized overnight in 25 mM Tris pH 8.0 containing 8 M Urea and 300 mM NaCl. Soluble protein was clarified by high speed centrifugation. The solubilized protein was added to Ni NTA Beads that were poured in Gravity Column and the column was washed with five column volumes of wash buffer (8 M urea, 20 mM Tris, pH 8.0, 300 mM NaCl, 20 mM imidazole) packed with Ni NTA. The protein was then eluted with buffer (8 M urea, 20 mM Tris, pH 8.0, 300 mM NaCl, 50-250 mM imidazole). The purity of VSIG3 protein was evaluated by SDS-PAGE. Human VSIG3 was refolded by drop-wise dilution into 25 mM Tris pH 8.0 containing 1 M L-Arg hydrochloride, 0.25 mM oxidized glutathione and 0.25 mM reduced glutathione. Superdex 75 (GE Healthcare, #10298948) equilibrated with 25 mM Tris pH 8.0 containing 150 mM NaCl was used to analyze the oligomeric state of each protein. The purified VSIG3 protein was used for crystallization studied.

### Expression of Seleno-Recombinant Proteins VSIG3

In order to obtain phase information and analyze the final crystal structure, *E. coil* cells were cultured in M9-deficient medium (Molecular Dimensions), and selenomethionine (Acros Organics) was added in the process of VSIG3 expression. The methionine in the final expressed VSIG3 protein was replaced by seleno methionine.

### Crystallization and Data Collection of Human VSIG3

Purified human VSIG3 was concentrated to 3 mg/mL. Diffraction-quality crystals were obtained at 4°Cfrom 0.1M sodium citrate tribasic dihydrate pH 5.6, containing 0.01 M Iron(III) chloride hexahydrate and 10% v/v Jeffamine M-600 using vapor diffusion method (hanging drop method). Diffraction data were collected on the BL18U beam line of the Shanghai Synchrotron Research Facility.

### Structure Determination and Refinement

The structure was determined using the molecular replacement method with PHASER in the CCP4 suite (31). Density map improvement by updating and refinement of the atoms was performed with ARP/wARP (32). Subsequent model building and refinement were performed using COOT and PHENIX, respectively (33, 34). All structure figures were generated with PyMol (35).

### The Purification of Human VSIG3 Protein Expressed by CHO Cells

The human VSIG3-ECD plasmid was cloned into the Spe I–Xho I sites of the vector pcDNA3.1+. We transfected plasmids into ExpiCHO cells (ThermoFisher, A29127). Supernatant was harvested on day 8 and purified by nickel column and competitively eluted with different concentrations of imidazole buffer (GenScript). Protein was concentrated using 10K MWCO spin columns (Merck). The purified protein was used for functional verification.

### Cell Culture and Reagents

PBMCs were purchased from Allcells Biotechnology (Shanghai) Co., LTD, and were used immediately after resuscitation and cannot be subcultured. Human CD4^+^ T cells were isolated from PBMCs using an EasySep™ human CD4^+^ T cell isolation kit (Stem Cell, Cat#17952) according to the manufacturer’s protocol, and cultured in complete RPMI-1640 media: RPMI 1640 medium (Biological Industries, Cat#01-100-1A) supplemented with 10% fetal bovine serum (Biological Industries, Cat#04-010-1A), 1% penicillin–streptomycin (Biological Industries, Cat#03-031-1B). All cell lines were regularly tested for mycoplasma contamination.

### *In Vitro* Plate-Bound T Cell Activation Assay

For PBMCs or human CD4^+^ T cell cultures, 96-well flat-bottom plates (ThermoFisher, Cat#AB0751) were coated with anti-human CD3 antibody (clone OKT3, Biolegend, Cat#317326) at 1 μg/mL mixed together with 0 μg/mL, 2.5 μg/ml, 5 μg/mL, 10μg/mL or 20μg/mL of the human VSIG3-ECD protein in PBS at 4°C overnight. The PBMCs or human CD4^+^ T cells were plated at a density of 1×10^5^ cells/well in complete RPMI medium. The cell supernatants were harvested after 48 h for the cytokine measurement by human IFN-γELISA kit (Biolegend, Cat#430104) and human TNF-αELISA kit (Biolegend, Cat#430204).

### Cell Proliferation Assay

PBMCs were labeled with 5-(and 6)-carboxyfluorescein diacetate succinimidyl ester (CFSE) following the manufacturer’s protocol (Biolegend, Cat#423801). Briefly, cells were labeled at 10^6^ cells per mL at 37°C for 10 minutes with 5 mmol/L CFSE in PBS containing 0.1% BSA. CFSE was quenched by adding twice the volume of complete media, followed by three washes in complete media. PBMCs were stimulated with anti-human CD3 antibody. Cell proliferation was analyzed on day 5 day by determining CFSE profiles using flow cytometry (BD Accuri C6).

### Co-Immunoprecipitation

HEK293T cells were transfected with FLAG tagged VSIG3 encoding plasmid using Lipo293™ (Beyotime Biotechnology, Nanjing, China) according to the manufacturer’s instructions. Forty-eight hours after transfection, cells were lysed in IP lysis buffer [10 mM Tris-HCl, 150 mM NaCl, 1 mM EDTA, 1% Triton, and 0.5% NP-40 (pH 7.5)] containing protease and phosphatase inhibitors for 30 min on ice with gentle rocking. The cell lysates were clarified after centrifugation and the supernatants were collected. Equal amounts of protein were subjected to incubation with Protein A/G magnetic beads (Cat#B23202, Bimake) pre-incubated with mouse antibodies against FLAG (Cat#AE005, Abclonal) or control immunoglobulin G (Cat#AC011, abclonal). Alternatively, cell lysates were overnight incubated with Anti-FLAG magnetic beads (magnetic beads covalently coupled with mouse anti-FLAG antibody) (Cat# B26102, Bimake). The immunocomplexes were subjected to Western blot analysis using antibodies against FLAG (Cat#AE004, Abclonal), HA (Cat#AE036, Abclonal) and GAPDH (Cat#ARG10112, Arigo).

### Visualization of Docked Ligands With the VSIG3 Protein

The VSIG3 PDB files were downloaded. All heterogeneous atoms were removed from VSIG3 for subsequent virtual screening. The VSIG3 docking grid was maximized for 80,000 virtual docking compounds. PDB file of VSIG3 was converted to the PDBQT format as macromolecules before virtual screening. The grid (ligand docking search space) was located as described above. Then, Autodock Vina 1.1.2 (36) was used for the subsequent molecular docking. After docking, docked compounds (binding energy < -6kcal/mol) located within the VSIG3/VISTA interface were selected as candidates for subsequent experimental verification. Protein–ligand interactions were visualized using Pymol version 1.7.4.5. The amino acid residues of VSIG3 protein close to the hit ligands (≤1 Å) were highlighted as potential interactive residues involved in the protein–ligand interaction.

### Microscale Thermophoresis (MST) Experiment

Human VSIG3-ECD was labeled according to the protocol provided in the Monolith NT™ Protein Labeling Kit RED-NHS (NanoTemper Technologies GmbH). Compounds were prepared in up to 16 serial dilutions and mixed with the labeled protein in the same volume. Then, the mixtures were incubated at room temperature for 30 min in the dark. Capillary forces were used to introduce the samples into the MST capillaries. The samples were placed on a tray that was inserted into the instrument. A fluorescence scan was performed across the capillaries to determine the position of the capillaries with micrometer precision. After this scan, up to 16 subsequent thermophoresis measurements were performed to determine the binding affinity.

### Activity Evaluation of Compound K204-3046 *In Vitro*

For anti-CD3 antibody induced PBMCs proliferation, 1 µg/mL anti-human CD3 antibody and 10 µg/mL human VSIG-3 ECD protein were coated in the 96-well plates overnight at 4°C. Then compound K204-3046 was added into each well containing 1×10^5^ PBMCs per well and cultured for 48h. The cell supernatant was collected, and the cytokine secretion level in the supernatant was detected by an ELISA kit. For the CSFE labeled PBMCs proliferation assay, CSFE-labeled PBMCs were incubated with plate-bound anti-human CD3 antibody (1 µg/mL), human VSIG-3 ECD protein (10 µg/mL), and compound K204-3046 at the indicated concentrations for 120 h. Cell proliferation was analyzed on day 5 day for CFSE profiles by flow cytometry (BD Accuri C6).

### Statistical Analysis

Student’s t-test was used for statistical analysis, and p-values reflect comparison with the control sample. P-values less than 0.05 were considered statistically significant. Data analysis and drawing was performed using Graphpad Prism 8.

## Results

### Overall Crystallization and Structure Determination of Human VSIG3-ECD Expressed in *E. coli*

**Figure 1:**
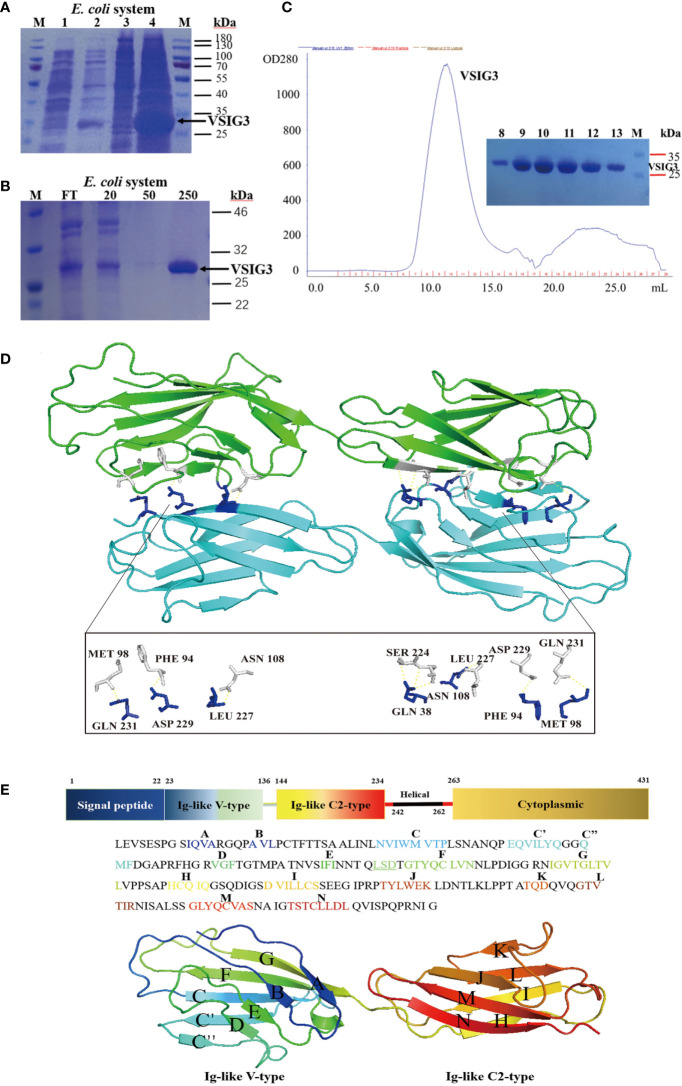
The crystal structure of VSIG3 protein. **(A)** Coomassie blue staining results of *E. coli* expressed VSIG3 protein. (1: before IPTG induction; 2: after IPTG induction; 3: supernatant after lysis; 4: inclusion bodies). **(B)** The VSIG3 protein was eluted with different concentrations of imidazole solutions (20 mM, 50 mM, 250 mM). FT: Flow through solutions. **(C)** Purification of refolded VSIG3 protein by size exclusion chromatography on Superdex 75. **(D)** Overview of the human VSIG3-ECD protein crystal structure as a dimer. Protein monomers interact with each other by hydrogen bonding. Chain A (green), chain B (cyan). **(E)** Detail of the structure of VSIG3-ECD protein. Sequence of human VSIG3-ECD with labeled secondary structure. Cartoon structure of human VSIG3-ECD with labeled secondary structure.

We produced recombinant extracellular domains of human VSIG3 in *E. coli*. The VSIG3-ECD protein was mainly found in inclusion bodies and was denatured and refolded to restore activity (**Figures 1A–C**). Crystals from *E. coli* expressing VSIG3-ECD were obtained using the hanging drop vapor diffusion method. The crystal structure of VSIG3 was resolved by X-ray diffraction. The final Ramachandran statistics are as follows: 92.12% favored, 7.40% allowed and 0.48% outliers for the final structure. The structure refinement statistics are listed in **Table 1**. The VSIG3 protein exists as a dimer, and monomers (chain A and chain B) are mainly connected by hydrogen bonding. MET108, PHE104, ASN118, SER234, LEU237, ASP239, and GLN241 of chain A VSIG3 protein interact with GLN241, ASP239, LEU237, GLN48, ASN118, PHE104, and MET108 of chain B VSIG3 protein (**Figure 1D**). The VSIG3 protein is a transmembrane protein consisting of four parts, including a 22-amino acid signal peptide, an extracellular domain consisting of an IgV segment and an IgC2 segment, a transmembrane domain consisting of 21 amino acids and an intracellular domain (**Figure 1E**).

**Table 1:** Data collection and refinement statistics.

	VSIG3
**Data collection**	
Space Group	*P*2_1_2_1_2_1_
Cell dimensions	
a, b, c (Å)	66.180, 87.065, 124.511
α, β, γ, (°)	90, 90, 90
Resolution (Å)	45.35~2.64
R_merge_ (%)	13.8 (75.8)
I/σI	9.05 (0.98)
Completeness (%)	97.6(91.4)
Redundancy	4.3 (3.2)
**Refinement**	
No. reflections	21,286
R_work_/R_free_ (%)	20.83/25.66
B-factors	
Protein	54.64
Water	51.11
R.m.s. deviations	
Bond lengths (Å)	0.009
Bond angles (°)	1.115
Ramachandran plot statistics (%)	
Most favored	92.12
Allowed	7.4
Disallowed	0.48

Values in parentheses are for the highest resolution shell.

Rmerge=ΣhΣi|Ih,i-Ih|/ΣhΣiIh,i, where Ih is the mean intensity of the i observations of symmetry related reflections of h.

R=Σ|Fobs-Fcalc|/ΣFobs, where Fcalc is the calculated protein structure factor from the atomic model (R_free_ was calculated with 5% of the reflections selected randomly).

### Functional Studies of Human VSIG3 Expressed in CHO Cells

**Figure 2:**
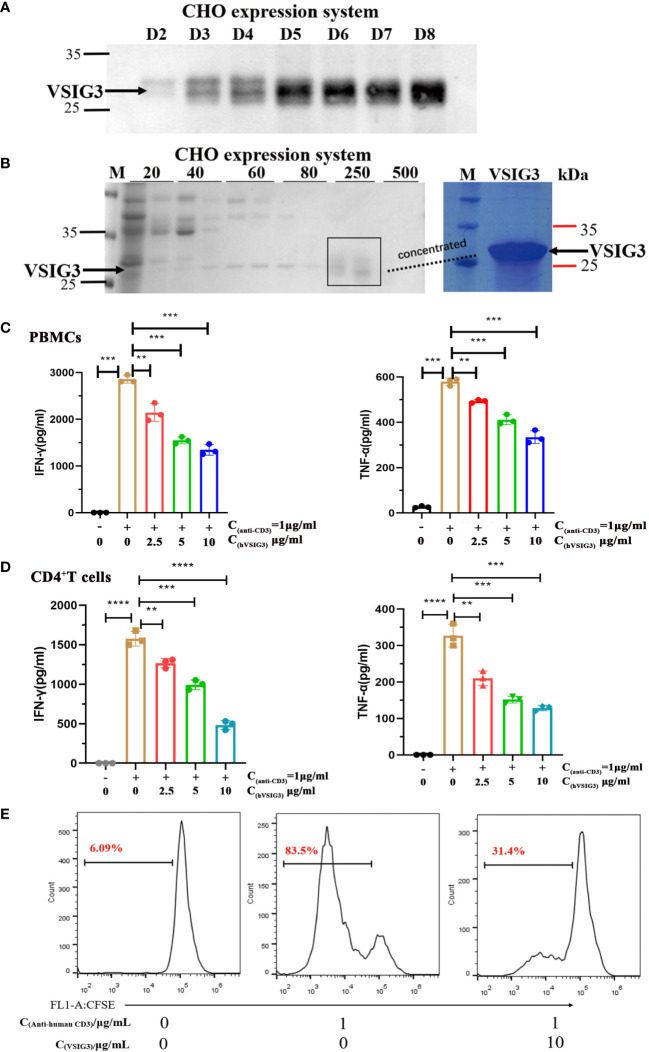
VSIG3 protein inhibits T cell function. **(A)** VSIG3 expression by CHO cells was detected by western blot after 2-8 days of culturing. **(B)** The VSIG3 protein was eluted with different concentrations of imidazole solutions (20 mM, 40 mM, 60 mM, 80 mM, 250 mM, 500 mM) (Left); Coomassie blue stained PAGE of concentrated VSIG3 protein (Right). **(C)** VSIG3 inhibits the cytokine secretion of activated PBMCs. Anti-human CD3 antibody (1 μg/mL) and different concentrations of VSIG3-ECD protein were coated on 96-well plates. PBMCs (1×10^5^ cells/well) were added for 48 hours to detect the secretion of IFN-γ and TNF-α in the supernatant. **p<0.01 and ***p<0.001 vs. control. **(D)** VSIG3 inhibits the secretion of IFN-γ and TNF-α in activated human CD4^+^ T cells. Anti-human CD3 antibody (1 μg/mL) and different concentrations of VSIG3-ECD protein were coated on 96-well plates. Human CD4^+^ T cells (1×10^5^ cells/well) were added for 48 hours to detect the secretion of IFN-γ and TNF-α in the supernatant. **p<0.01, ***p<0.001 and ****p<0.0001 vs. control. **(E)** VSIG3 inhibits the proliferation of activated PBMCs. CFSE-labeled PBMCs (1×10^5^ cells/well) were stimulated by plate-bound anti-human-CD3 (1 μg/mL), and VSIG3 protein (10 μg/mL) was added. The proliferation levels of PBMCs were measured by flow cytometry on day 5. Representative results from three independent experiments are shown.

We also expressed and purified VSIG3 using a CHO-based expression system. The histidine-tagged target protein in CHO cell supernatants was eluted from Ni NTA beads with 250 mM imidazole (**Figures 2A, B**). Anti-human CD3 antibody (1 µg/mL) and human VSIG3-ECD protein expressed by CHO cells were coated at different concentrations in 96-well plates at 4°C overnight, and then peripheral blood mononuclear cells (PBMCs) were added (1×10^5^ cells/well) and cultured for 48 h. The secretion levels of cytokines IFN-γ and TNF-α in the supernatant were detected using ELISA kits. The results showed that VSIG3 significantly inhibited the secretion of IFN-γ and TNF-α by PBMCs in a dose-dependent manner (**Figure 2C**). Similarly, VSIG3 significantly inhibited the secretion of IFN-γ and TNF-α in human CD4^+^ T cells (**Figure 2D**). To further demonstrate the biological function of VSIG3, we evaluated the effect of CHO-expressed VSIG3 (10 μg/mL) on the proliferation of activated PBMCs (1×10^5^ cells/well). Anti-human CD3 antibody (1 μg/mL) was added to a 96-well plate, and CFSE-labeled PBMCs were cultured. Proliferation was measured on day 5 by flow cytometry. The results showed that VSIG3 has a biological function of inhibiting the proliferation of PBMCs (**Figure 2E**).

### Protein-Protein Docking for VISTA and VSIG3

**Figure 3:**
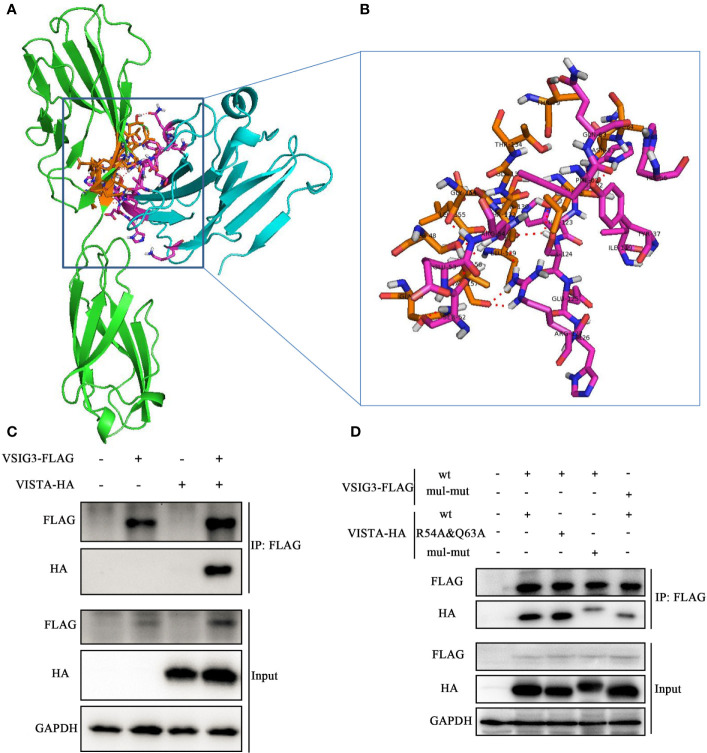
Protein-protein docking for VISTA and VSIG3. **(A)** An overall map of the virtual docking of the human VISTA ECD (cyan) with the human VSIG3 ECD protein (green) based on the structures of hVISTA and hVSIG3. Key residues are depicted in stick representation (purple and orange). **(B)** Enlarged the schematic diagram of the binding site. **(C)** HEK293T cells were transfected with VSIG3-FLAG and VISTA-HA plasmids. Equal amounts of protein were immunoprecipitated with Anti-FLAG magnetic beads and immunoblotted with antibodies indicated. **(D)** Co-IP detected the interaction by multiple mutated amino acid epitope on VISTA and VSIG3. HEK293T cells were transfected with wild type (wt) or multiple point mutations (mul-mut) of VSIG3-FLAG or VISTA-HA plasmids. Equal amounts of protein were immunoprecipitated Anti-FLAG magnetic beads and immunoblotted with antibodies indicated.

To address the interaction between VISTA and VSIG3, we used ClusPro, which generated 30 protein-protein docking models for VISTA (PDB: 6OIL) and VSIG3. Structural alignment shows that the best protein-protein docking model is similar to PD-1/PD-L1 (PDB: 4ZQK). This result suggests that the VISTA/VSIG3 complex is similar to the PD-1/PD-L1 complex. The final protein-protein interaction structure for VISTA and VSIG3 is shown in **Figures 3A, B**.

In addition, we cotransfected a FLAG-tagged VSIG3-encoding plasmid and an HA-tagged VISTA-encoding plasmid into HEK293T cells and determined the binding of VISTA and VSIG3 by immunoprecipitation (**Figure 3C**). This result indicates that the VSIG3/VISTA interaction is biologically relevant and may represent a new independent pathway related to the classic B7 family. Amino acids potentially important for the interaction between VISTA and VSIG3, as predicted by bioinformatics, were mutated to alanine, and the interaction of VISTA with VSIG3 was lost, as observed by Co-IP experiments. This result indicates that the VSIG3/VISTA interaction is biologically relevant. Multiple point mutations (mul-mut) of VSIG3 or VISTA decreased their interaction (**Figure 3D**). The mutants are as follows: VISTA mutations include K2A, Y37A, S52A, E53A, R54A, F62A, Q63A, H66A, I119A, HHSEHR122−127AAAAAA and S152A; VSIG3 mutations include S7A, Q9A, LSN40−42AAA, LSDTGT88−93AAAAAA and GLTV113−116AAAA.

### Computational Virtual Screening for VSIG3 Small-Molecule Hits

**Figure 4:**
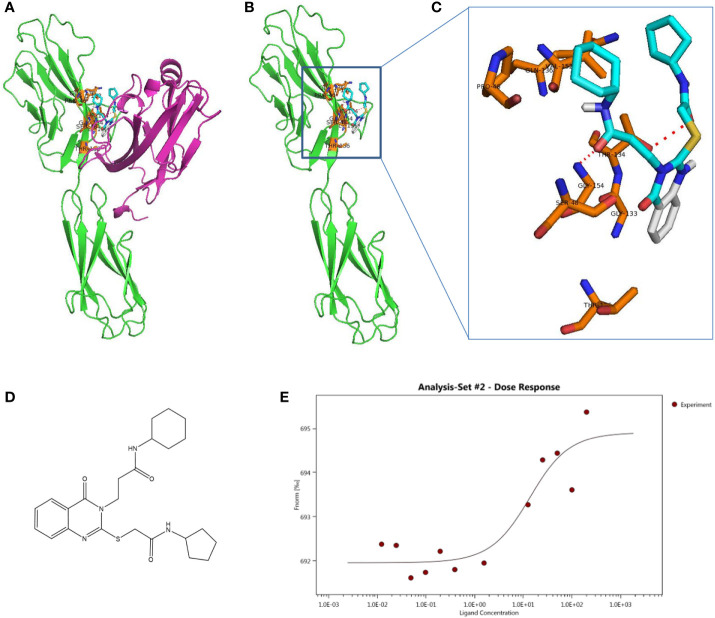
Schematic diagram of docking structure of small molecule compound K284-3046 and VSIG3 protein. **(A)** K284-3046 binds to VSIG3/VISTA interface. K284-3046 (blue stick) docked superposition on VSIG3 (green)/VISTA (purple) interface. **(B)** Interactive amino acids (orange sticks) with K284-3046 (blue stick) on VSIG3 (red dash line indicates the hydrogen bonds). **(C)** VSIG3 protein extracellular domain and the docked positions of K284-3046. **(D)** The structure of compound K284-3046. **(E)** The MST assay evaluates the KD value of K284-3046 binding to VSIG3 protein. The KD value is 11.61 ± 9.12 μM.

To identify small molecular compounds binding to VSIG3, VSIG3 crystal structure files were applied for computational screening. All heterogeneous atoms were removed from VSIG3 for subsequent virtual screening. The VSIG3 docking grid was maximized for 80,000 virtual compounds. Approximately 80,000 SDF files of structurally diverse small molecules from the Chemdiv library were converted to PDBQT format as ligands using Open Babel (version 2.4). The grid (ligand docking search space) was maximized based on VSIG3-ECD. After docking, compounds (binding energy < -6 kcal/mol) located within the VSIG3/VISTA interface were selected as candidates for subsequent experimental verification. Protein–ligand interactions were visualized using PyMOL version 1.7.4.5. The amino acid residues of the VSIG3 protein close to the hits (≤1 Å) were highlighted as potential residues involved in the protein–ligand interaction. Ten candidate compounds were selected to determine their binding rates with VSIG3-ECD by ELISA. The top five small molecules were identified as lead compounds with a binding rate of greater than 10% (**Table S1**). Next, the binding characteristics of the five compounds to VSIG3 were assayed by microscale thermophoresis (MST). K204-3046 and VSIG3 had a KD value of 11.61 ± 9.12 μM in the MST assay, demonstrated that K204-3046 has strong affinity with the human VSIG3 protein (**Figures 4D, E**). The interactions of the human VSIG3 protein and its ligand K204-3046 were visualized using PyMOL software. K284-3046 binds to amino acids PRO46, SER48, GLY133, THR134, GLN136, VAL152, GLY154 and THR156 of VSIG3 (**Figures 4A–C**). In subsequent experiments, we chose K204-3046 to verify its biological activities on VSIG3.

### Compound K204-3046 Inhibits VSIG3 Function on T Cells

**Figure 5:**
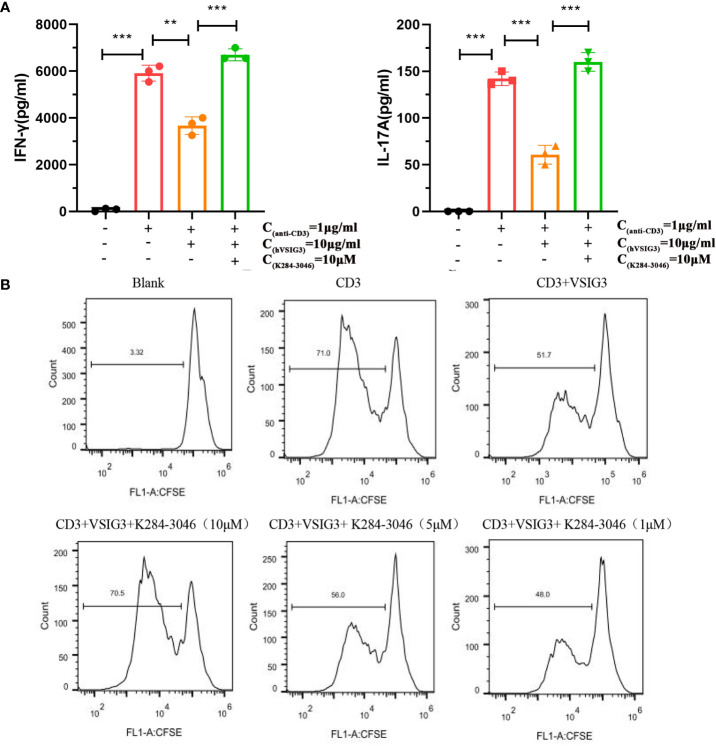
K284-3046 blunted the inhibitory effect of VSIG3 on activated PBMCs and improved the inhibitory effect of VSIG3 on the proliferation of PBMCs. **(A)** A total of 1×10^5^ PBMCs were incubated with an immobilized anti-human CD3 antibody (1 μg/mL) and human VSIG3 at 10 μg/mL, and compound K204-3046 was added as indicated. The levels of IFN-γ and IL-17 in the cell culture supernatants were measured at 48 h using ELISA kits. ns: not significant, **p<0.01 and ***p<0.001 vs. control. **(B)** A total of 1×10^5^ CFSE-labeled PBMCs were stimulated with plate-bound anti-human CD3 (1 μg/mL) together with co-absorbed human VSIG3 protein at 10 μg/mL, and compound K204-3046 was added at 10 μM. The proliferation levels of PBMCs were measured. Representative results from three independent experiments are shown.

PBMCs secrete large amounts of IL-17 and IFN-γ when stimulated with an anti-human CD3 antibody. IL-17 and IFN-γ levels are reduced after adding CHO-expressing VSIG3 protein. Moreover, cytokine levels were increased after K284-3046 was added (**Figure 5A**). Furthermore, VSIG3 suppressed PBMC proliferation in response to anti-human CD3 stimulation. PBMC proliferation was promoted after adding K284-3046 (**Figure 5B**). Therefore, K284-3046 inhibits the function of the human VSIG3 protein and may be an inhibitor of VSIG3.

## Discussion

Targeting the programmed cell death protein 1 (PD-1)/programmed cell death 1 ligand 1 (PD-L1) immunological checkpoint with monoclonal antibodies has provided remarkable success in cancer treatment in recent years (37). However, less than 30% of patients may benefit from this treatment. Novel immune checkpoints or coinhibitory pathways need to be identified (38–41). VISTA is a novel immunological checkpoint molecule belonging to the immunoglobulin family, and VSIG3 is highly expressed in intestinal cancer as a tumor-associated antigen. VSIG3 was reported as one of the potential ligands for VISTA (26, 27). Thus, the identification or synthesis of VSIG3 inhibitors, preferably antibodies targeting VSIG3 and/or the VSIG3/VISTA interaction, is important in the treatment of cancer. The development of inhibitors of the VISTA/VSIG3 pathway lags behind antibody development due to insufficient structural information.

In this study, we present the crystal structure of the extracellular domain of the human VSIG3 protein produced in *E. coli* at 2.64-angstrom resolution. To our knowledge, this is the first time a high-resolution structure of VSIG3 has been reported (**Figure 1**; **Table 1**). Additionally, using CHO-produced human VSIG3 ECD, we demonstrated that VSIG3 acts as an inhibitory ligand, as concluded from data on T cell proliferation and T cell cytokine secretion profiles (**Figure 2**; **Figure S1**). These findings are consistent with previous literature reports (27).

Mehta et al. recently reported a 1.85-angstrom crystal structure of the elusive human VISTA extracellular domain by a combinatorial MR-Rosetta approach, highlighting its two additional disulfide bonds and protruding C–C’ loop compared to the other B7 family members (42). A pH-dependent binding interaction was recently identified between VISTA and PSGL-1, a receptor expressed on leukocytes that plays a role in immune cell trafficking (28). In addition, these researchers observed moderate pH-selective binding of VISTA to VSIG-3 using the Octet biosensor but could not detect specific binding in cell-based assays and observed no competition between VSIG-3 and PSGL-1. Mehta et al. also found that binding to VISTA and PSGL-1 was heavily dependent on the acidic pH as assessed by ELISA, whereas VSIG3 had a fourfold improvement in binding affinity at physiological pH (43). Here, we demonstrate the specific interaction between VSIG3 and VISTA by Co-IP and enzyme-linked immunosorbent assay (ELISA) (**Figure 3C**; **Figure S2**).

The anti-VISTA antibody VSTB112 inhibited VISTA signaling *in vitro* and resulted in tumor regression in a bladder cancer model using a human VISTA knock-in mice. Mehta et al. noted that the mapped VISTA epitope (54A/62A/63A) is not only important for interaction with VSTB112 but also involved in binding to VSIG3 (37). In addition, these researchers reported the use of yeast surface display to engineer an anti-VISTA antibody (SG7), which inhibits VISTA function and blocks purported interactions with both PSGL-1 and VSIG3 proteins. They found that mutations in a number of VISTA residues affected PSGL-1 binding (R54, F36, K38, T39, H121, H122, and Q63), and mutations in multiple VISTA residues significantly decreased binding to VSIG3 (F36, Y37, K38, T39, H122, R54, and Q63) (43). Here, we mutated the two amino acid epitopes of VISTA (R54 and Q63) and found that the binding of VISTA-VSIG3 did not change. On the other hand, we mutated multiple amino acid epitopes on VISTA and VSIG3 and observed decreased binding in Co-IP (**Figure 3**). Furthermore, based on the protein-protein docking study of VISTA/VSIG3, we report a small molecule inhibitor of VSIG3 K284-3046 and evaluated its biological activities *in vitro* (**Figures 4**, **5**; **Table S1**).

In summary, we reported here for the first time the crystal structure of the human VSIG3-ECD protein and further demonstrated the binding of VSIG3 to VISTA. Importantly, a small molecule inhibitor of VSIG3 K284-3046 was identified. This study provides the structural basis for designing antibodies or compounds for the unique VSIG3/VISTA coinhibitory pathway in the treatment of cancer or autoimmune diseases. Additionally, the coordinates for the VSIG3 ECD structure will also expedite cocrystallization efforts of VSIG3 complexes by providing a well-suited template for molecular replacement.

## Data Availability Statement

The raw data supporting the conclusions of this article will be made available by the authors, without undue reservation, to any qualified researcher.

## Author Contributions 

JL, HS, JJ, and XX designed the research. XX, CC, WC, LW, and TL performed the experiments. XX, CC, and WC analyzed the data. JL, XX, and WC wrote the paper. All authors contributed to the article and approved the submitted version.

## Funding

The paper was supported by the National Natural Science Foundation of China (No. 81973361) and Natural Science Foundation of Jiangsu Province (BK20202009). The authors thank Dr. Shilong Fan from Technology Center for Protein Science, Tsinghua University for assistance in determining the crystal structure.

## Conflict of Interest

The authors declare that the research was conducted in the absence of any commercial or financial relationships that could be construed as a potential conflict of interest.

## Supplementary Material

Supplementary Figure 1 Anti-CD3 antibody coating is unaffected by mixing in VSIG3 protein as determined by VSIG3 inhibited cytokine production by PBMCs. **(A)** The biotinylated anti-human CD3 antibody (1 μg/mL) and different concentrations of human VSIG3 protein were coated on 96-well flat bottom plates at 4°C for 16 hours. Binding of biotinylated anti-human CD3 was detected by adding streptavidin-HRP followed by substrate color reagents. 1×10^5^ PBMCs were stimulated with plate-bound anti-CD3 antibody in the presence of VSIG3 or control-IgG at a ratio of 1:2 (1 µg/ml CD3 and 2 µg/ml VSIG3 or control-Ig), 1:5, 1:10, 1:20. Culture supernatants were collected at 48 h, and the level of IFN-γ **(B)** and TNF-α **(C)** was analyzed by ELISA. Representative results from three independent experiments are shown.

Supplementary Figure 2 VSIG-3 binds to VISTA by ELISA. **(A)** The VISTA protein neutralizes the inhibitory effect of VSIG3 protein expressed by CHO cells on PBMCs activation. Exogenous VISTA was incubated with VSIG3 coated on 96-well plate, and then PBMCs were added. The expression levels of IFN-γ and TNF-α in PBMCs were evaluated with ELISA. *p<0.05, **p<0.01, ***p<0.001 and ****p<0.0001 vs. control. **(B)** Human VSIG3-ECD expressed by CHO cells specifically binds to recombinant VISTA in a functional ELISA binding assay. **(C)** Different concentrations of VSIG3 antibody inhibited the binding of VSIG3/VISTA. Representative results from three independent experiments are shown.

Supplementary Table 1 Candidate compound binding rate to VSIG3 protein
