# Supplementary figures and images for: Structural Basis of VSIG3: The Ligand for VISTA
Source: Front Immunol. 2021 Mar 25;12:625808. doi: 10.3389/fimmu.2021.625808 (PMC8027081; doi:10.3389/fimmu.2021.625808)

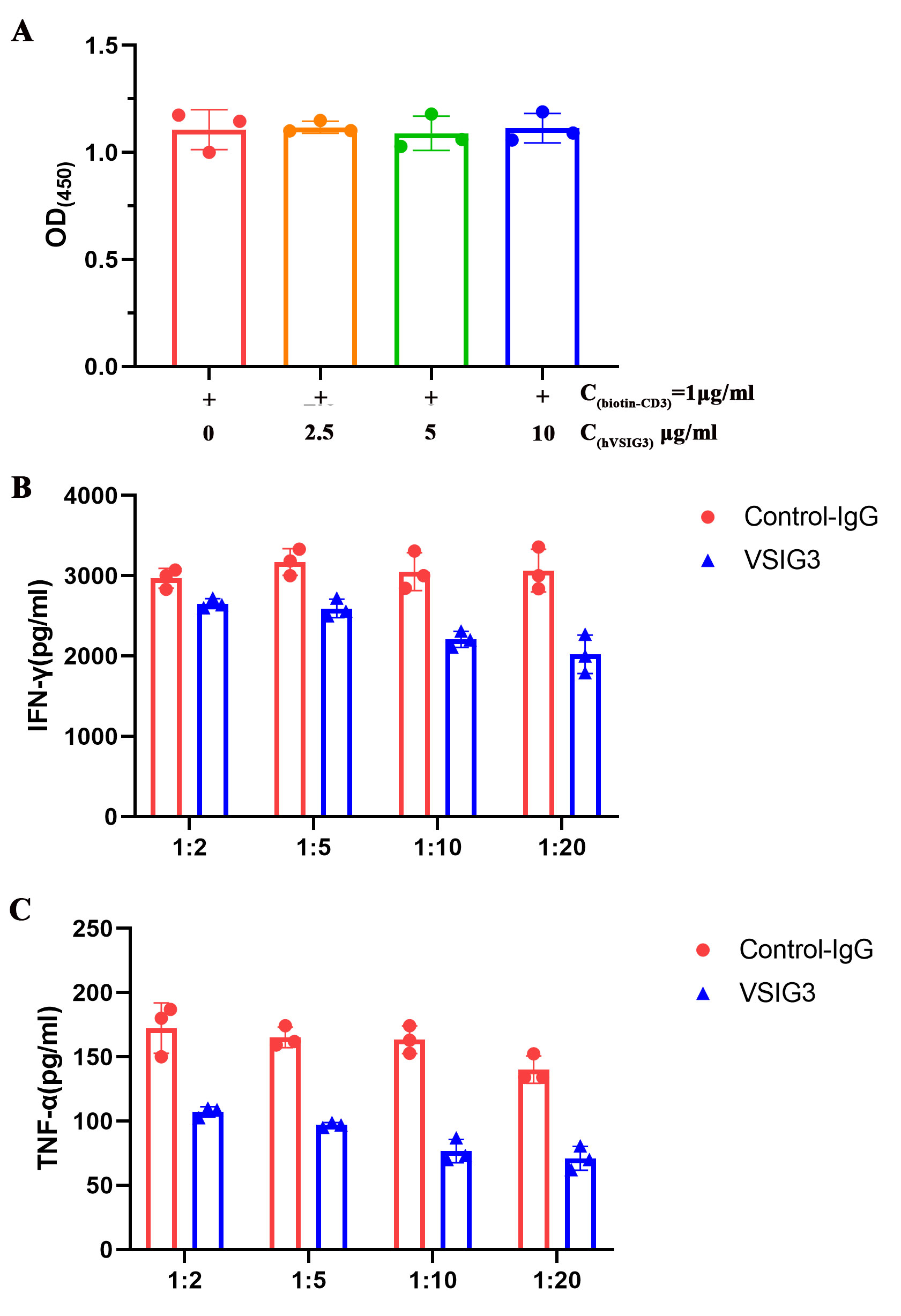

Supplement: Supplementary Figure 1 — Anti-CD3 antibody coating is unaffected by mixing in VSIG3 protein as determined by VSIG3 inhibited cytokine production by PBMCs. (A) The biotinylated anti-human CD3 antibody (1 μg/mL) and different concentrations of human VSIG3 protein were coated on 96-well flat bottom plates at 4°C for 16 hours. Binding of biotinylated anti-human CD3 was detected by adding streptavidin-HRP followed by substrate color reagents. 1×10^5^ PBMCs were stimulated with plate-bound anti-CD3 antibody in the presence of VSIG3 or control-IgG at a ratio of 1:2 (1 µg/ml CD3 and 2 µg/ml VSIG3 or control-Ig), 1:5, 1:10, 1:20. Culture supernatants were collected at 48 h, and the level of IFN-γ (B) and TNF-α (C) was analyzed by ELISA. Representative results from three independent experiments are shown. [file Image_1.jpeg]

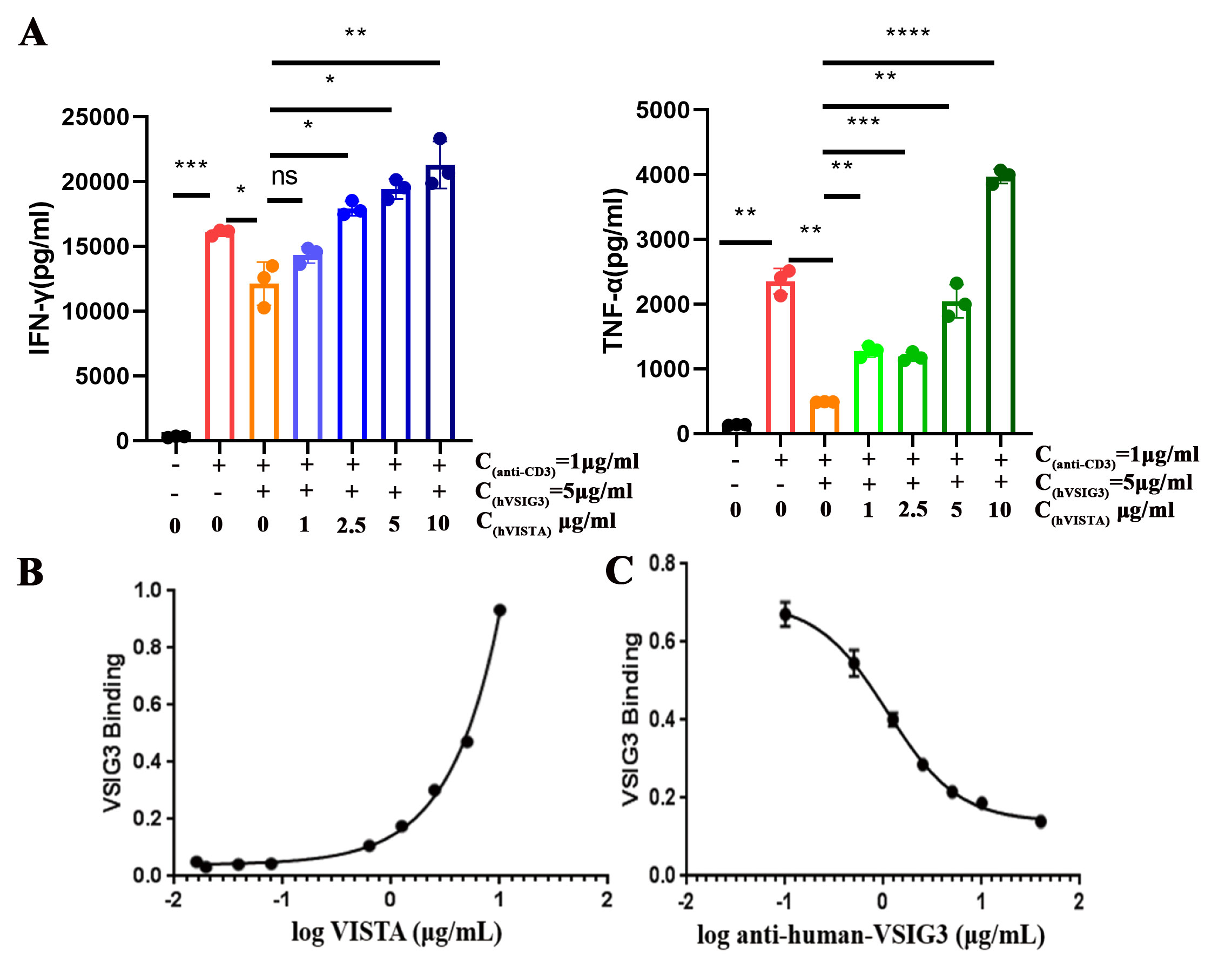

Supplement: Supplementary Figure 2 — VSIG-3 binds to VISTA by ELISA. (A) The VISTA protein neutralizes the inhibitory effect of VSIG3 protein expressed by CHO cells on PBMCs activation. Exogenous VISTA was incubated with VSIG3 coated on 96-well plate, and then PBMCs were added. The expression levels of IFN-γ and TNF-α in PBMCs were evaluated with ELISA. *p<0.05, **p<0.01, ***p<0.001 and ****p<0.0001 vs. control. (B) Human VSIG3-ECD expressed by CHO cells specifically binds to recombinant VISTA in a functional ELISA binding assay. (C) Different concentrations of VSIG3 antibody inhibited the binding of VSIG3/VISTA. Representative results from three independent experiments are shown. [file Image_2.jpeg]
