# Supplementary material for: Structural Basis of VSIG3: The Ligand for VISTA
Source: Front Immunol. 2021 Mar 25;12:625808. doi: 10.3389/fimmu.2021.625808 (PMC8027081; doi:10.3389/fimmu.2021.625808)
Supplement: Supplementary Table 1 — Candidate compound binding rate to VSIG3 protein [file Table_1.docx]

**Table S1. Candidate compound binding rate to VSIG3 protein**

| CAS Number | Binding rate | Structure |
| --- | --- | --- |
| L103-0670 | 19.31% |  |
| E894-0141 | 13.39% |  |
| G311-0333 | 12.87% |  |
| 5276-0950 | 10.15% |  |
| K284-3046 | 10.09% |  |
| E238-0006 | 9.29% |  |
| C800-0619 | 9.23% |  |
| E224-0024 | 8.93% |  |
| E956-0170 | 7.94% |  |
| C301-8707 | 7.86% |  |
