# Supplementary material for: Structural Basis of VSIG3: The Ligand for VISTA
Source: Front Immunol. 2021 Mar 25;12:625808. doi: 10.3389/fimmu.2021.625808 (PMC8027081; doi:10.3389/fimmu.2021.625808)
Supplement: Supplementary file 4 [file DataSheet_1.docx]

**Supplemental Methods and Materials**

**Control experiments**

Different concentrations of human VSIG3 protein (2.5 μg/mL, 5 μg/mL, 10 μg/mL) was coated on 96-well flat bottom plates at 4℃ for 16 hours. 1 μg/mL [biotinylated](../../Administrator/AppData/Local/youdao/dict/Application/8.9.5.0/resultui/html/index.html#/javascript:;) anti-human CD3 antibody (Biolegend, Cat#317320) was added at room temperature for 30 minutes with shaking. Binding of [biotinylated](../../Administrator/AppData/Local/youdao/dict/Application/8.9.5.0/resultui/html/index.html#/javascript:;) anti-human CD3 was detected by adding streptavidin-HRP (Biolegend, Cat#405210) followed by substrate color reagents (R&D system, Cat#DY999). The absorbance at 450nm was measured by Thermo Scientific Microplate Reader.

**Functional ELISA binding assay**

Recombinant human VSIG3 protein (2 µg/mL) was immobilized on 96-well ELISA plates (ThermoFisher, Cat#437796) by incubation at 2-8°C for 24 h. Then, ELISA plates were blocked with 1% BSA-PBS at room temperature for 2 h. Biotinylated human VISTA protein (R&D system, Cat#BT7126-050) at the indicated concentrations was subsequently added to each well of the ELISA plate and incubated at room temperature for 2 h. Bound VISTA was detected by adding streptavidin-HRP (Biolegend, Cat#405210) followed by substrate color reagents (R&D system, Cat#DY999).

**Supplemental Results**

We demonstrate that the anti-CD3 antibody coating is unaffected by mixing in VSIG3 protein using ELISA (Figure S1A), and VSIG3-Ig but not control-Ig suppressed the cytokines secretion of PBMCs in response to anti-CD3 stimulation (Figure S1B-S1C). We also used ELISA to confirm the interaction between VISTA with VSIG3. Our results showed that exogenous VISTA (more than 1μg/ml) neutralizes the inhibitory effect of VSIG3 protein on PBMCs activation (Figure S2A), which is similar to what was reported in the literature ^[27]^. As shown in Figure S2B, VISTA has a certain specific binding with VISTA but is weak. To further confirm the specific interaction between VISTA and VSIG-3, we carried out functional ELISA binding assays in the presence of anti-human VSIG3 antibodies (R&D systems). Inclusion of anti-human VSIG3 antibodies completely blocked this interaction, suggesting that VSIG-3 is a novel binding partner for B7 family member VISTA (Figure S2C).

**Supplemental figure and table legends**

**Table S1. Candidate compound binding rate to VSIG3 protein**

**Figure S1. Anti-CD3 antibody coating is unaffected by mixing in VSIG3 protein as determined by VSIG3 inhibited cytokine production by PBMCs.** (A) The [biotinylated](../../Administrator/AppData/Local/youdao/dict/Application/8.9.5.0/resultui/html/index.html#/javascript:;) anti-human CD3 antibody (1 μg/mL) and different concentrations of human VSIG3 protein were coated on 96-well flat bottom plates at 4℃ for 16 hours. Binding of [biotinylated](../../Administrator/AppData/Local/youdao/dict/Application/8.9.5.0/resultui/html/index.html#/javascript:;) anti-human CD3 was detected by adding streptavidin-HRP followed by substrate color reagents. 1×10^5^ PBMCs were stimulated with plate-bound anti-CD3 antibody in the presence of VSIG3 or control-IgG at a ratio of 1:2 (1 µg/ml CD3 and 2 µg/ml VSIG3 or control-Ig), 1:5, 1:10, 1:20. Culture supernatants were collected at 48 h, and the level of IFN-γ (B) and TNF-ɑ (C) was analyzed by ELISA. Representative results from three independent experiments are shown.

**Figure S2. VSIG-3 binds to VISTA by ELISA.** (A) The VISTA protein neutralizes the inhibitory effect of VSIG3 protein expressed by CHO cells on PBMCs activation. Exogenous VISTA was incubated with VSIG3 coated on 96-well plate, and then PBMCs were added. The expression levels of IFN-γ and TNF-ɑ in PBMCs were evaluated with ELISA. *p<0.05, **p<0.01, ***p<0.001 and ****p<0.0001 vs. control. (B) Human VSIG3-ECD expressed by CHO cells specifically binds to recombinant VISTA in a functional ELISA binding assay. (C) Different concentrations of VSIG3 antibody inhibited the binding of VSIG3/VISTA. Representative results from three independent experiments are shown.
